# Hypoglycemic Events Focusing on Situational Factors, Bystander Identification, and Prehospital Management

**DOI:** 10.3390/jcm15072746

**Published:** 2026-04-05

**Authors:** Asami Okada, Shiruku Watanabe, Yasuaki Koyama, Ryosuke Nomura, Tadahiro Goto

**Affiliations:** 1 TXP Medical Co., Ltd., Chiyoda 101-0025, Tokyo, Japan; 2 Department of Environmental Health Science and Public Health, Akita University Graduate School of Medicine, Akita-shi 010-8543, Akita, Japan; 3 Department of Emergency and Critical Care Medicine, Hitachi General Hospital, Hitachi 317-0077, Ibaraki, Japan; 4 Department of Emergency Medicine, South Miyagi Medical Center, Ogawara 989-1253, Miyagi, Japan; 5 Department of Health Data Science, Graduate School of Data Science, Yokohama City University, Yokohama 236-0027, Kanagawa, Japan

**Keywords:** severe hypoglycemia, prehospital intervention for hypoglycemia, diabetes mellitus, hypoglycemic episode, insulin

## Abstract

**Background:** Severe hypoglycemia is a major reason for emergency medical service (EMS) activation among patients with diabetes. However, real-world epidemiology, including onset location, timing, caller identity, and prehospital management, remains insufficiently described. This study aimed to characterize these cases and assess prehospital interventions and patient outcomes. **Methods:** We conducted a retrospective, descriptive study using EMS transport records and emergency department (ED) data from two core hospitals and their regional EMS systems in Japan between January 2018 and December 2023. Included patients were those transported by EMS for hypoglycemia with a corresponding ED diagnosis. Extracted data included patient characteristics, episode location and time, EMS caller identity, prehospital interventions, and clinical outcomes. **Results:** Among 237 episodes, the median age was 74 years and 59.9% were male. Most events occurred at home (78.1%) and during evening or nighttime hours (51.9%). Family members were the most frequent EMS callers (67.5%), yet 12.5% of patients received bystander medical intervention. EMS teams performed most prehospital interventions (68.8%), primarily intravenous glucose administration (65.2%). At EMS arrival, 16.0% were fully conscious and 21.1% were comatose. Hospitalization occurred in 44.3%. The hospitalization rate was 34.2% among patients who received prehospital intervention and 53.2% among those who did not. **Conclusions:** Most hypoglycemia episodes were discovered by family members, but bystander intervention was uncommon. Differences in hospitalization rates were observed according to the presence and timing of prehospital intervention.

## 1. Background

Severe hypoglycemia remains a significant clinical concern in the management of diabetes, particularly among individuals receiving insulin or insulin secretagogues. In addition to its acute neurological manifestations, severe hypoglycemia is associated with cardiovascular complications, impaired quality of life, and increased healthcare utilization. While its acute manifestations, ranging from neuroglycopenic symptoms and loss of consciousness to seizures and, in rare cases, irreversible neurological damage, are well recognized, the real-world circumstances under which hypoglycemic episodes occur, particularly those severe enough to warrant emergency medical service (EMS) activation, are less systematically described [1,2].

These temporal and environmental patterns suggest a confluence of behavioral, pharmacologic, and social factors, such as mismatches between insulin action and carbohydrate intake, reduced glucose monitoring during sleep, and caregiver presence, that contribute to the recognition and escalation of hypoglycemic events. In the U.S. and Europe, severe hypoglycemia has been estimated to account for 4–10% of mortality in type 1 diabetes [3,4] and is a leading cause of hospitalization among insulin-treated type 2 diabetes patients [1,5]. However, most existing evidence originates from Western populations, and data describing the situational context of hypoglycemia requiring EMS activation remain limited [6,7,8].

Despite these insights, there remains a lack of detailed regional data from Japan, where differences in population demographics, cultural back grounds, healthcare infrastructure, and EMS organization may significantly alter the context in which hypoglycemia occurs. Japan’s aging population and the high proportion of elderly individuals with diabetes, coupled with centralized EMS systems and hospital referral pathways, may influence not only the frequency and severity of episodes but also the likelihood of detection and timely treatment [9].

The two regions are characterized by slightly higher proportions older adults compared with the national average. Hitachi City has a population of approximately 185,000, while the Sennan region has approximately 166,000 residents. The proportion of individuals aged 65 years or older in these regions exceeds the national average (Hitachi: 34%, Sennan: 35%, Japan: 28%), which may influence the incidence and management of hypoglycemia-related EMS transports [10,11].

This study aims to characterize the demographic and clinical profiles of patients experiencing hypoglycemia-related EMS activations, describe the settings and contexts in which these episodes arise, and describe the nature and timeliness of prehospital interventions administered by EMS personnel and bystanders in Japan. By providing descriptive data from two regional healthcare systems, this study seeks to contribute to a better understanding of hypoglycemia-related EMS utilization in regional healthcare systems in Japan.

## 2. Methods

### 2.1. Study Design and Setting

This study is a retrospective, descriptive study using EMS transport records and emergency department (ED) records from two regional EMS systems in Japan and their corresponding core hospitals. The hospitals, Hitachi General Hospital in Ibaraki Prefecture and Miyagi Prefectural South Hospital in the Sennan region of Miyagi Prefecture, serve as central medical institutions within their respective areas and are representative of the local healthcare systems. These sites were selected because EMS transports in each region are largely centralized to these core hospitals, allowing for regionally representative data collection.

All measurements were extracted from structured fields within EMS transport records and the electronic medical records based on the NEXT Stage ER (NSER; TXP Medical Co., Ltd., Tokyo, Japan) system at participating hospitals. Data extraction followed standardized procedures to ensure consistency across study sites. In the exploratory phase of the analysis, the study also compared clinical outcomes according to the presence or absence of prehospital intervention.

### 2.2. Data Sources

This study used EMS transport records maintained by the fire departments in the selected regions and electronic medical records from the participating hospitals’ ED. EMS records provide precise time-related information, including response times, on-scene times, and transport times, while ED records include detailed patient conditions, interventions, and outcomes. Data extraction follows standardized protocols to ensure consistency and reliability. All data will be pseudonymized before analysis, and individual patient identifiers will not be shared outside the study sites.

The NSER is an electronic medical record interface designed to assist practitioners in recording and structuring clinical data [12,13]. Through the NSER, physicians and nurses input patient information into predefined fields, including chief complaints, present illness, medical history, physical assessments, and ED diagnoses. For chief complaints, practitioners can select from a predefined list or enter free-text descriptions when necessary. These complaints are automatically categorized into 231 chief complaint categories based on the Japan Triage and Acuity Scale, which is derived from the Canadian Triage and Acuity System [14].

The NSER also records medical history and physician diagnoses, mapping them to ICD-10 codes, and encodes medication data using the WHO Anatomical Therapeutic Chemical Classification System. The system is designed for data structuring and clinical research purposes and does not provide clinical decision support or diagnostic suggestions. Importantly, the system does not require legal authorization as a medical device, as confirmed by the Tokyo Metropolitan Pharmaceutical Affairs Bureau.

### 2.3. Study Participants

The study included patients who were transported by EMS to participating hospitals for suspected or confirmed hypoglycemia between January 2018 and December 2023 and had a documented hospital evaluation.

The inclusion criteria for this study required that patients be registered in the NSER system at participating hospitals. Additionally, patients had to have a diagnosis code (ICD-10: E15 or E16) indicating hypoglycemia. Furthermore, the study included patients whose diagnosis text explicitly mentioned “hypoglycemia.” Lastly, patients whose medical history or clinical notes contained terms suggesting hypoglycemia, such as “hypoG,” “hypoglycemia,” “low BG,” “low BS,” “glucagon administration,” or “glucose administration,” were also eligible for inclusion. Inclusion was not based solely on glucose administration but required diagnostic coding or documentation consistent with hypoglycemia. Medication use was determined based on structured outpatient medication fields in the NSER system. Only medications recorded and successfully mapped to standardized drug dictionaries were classified as antidiabetic agents. Absence of documentation in this field was treated as no documented medication use.

The exclusion criteria for this study included patients who requested EMS but were not transported to a hospital. Additionally, patients who were transported by EMS but were not recorded in the NSER system were excluded. Furthermore, patients who were transported by EMS but were not taken to the designated regional hospitals were also not eligible for inclusion in the study. Patients with cardiac arrest at hospital arrival were also excluded, according to a previous study [15].

### 2.4. Measurements

This study collected detailed information on both the prehospital and in-hospital phases of EMS transports related to hypoglycemia. Variables obtained from EMS records included the location where the patient was found (e.g., home or public place), the estimated time of symptom onset, the person who identified the patient and called for EMS, and time-related metrics such as the time from EMS call to scene arrival, time spent on scene, and time from call to hospital arrival. The presence or absence of prehospital interventions such as glucose or glucagon administration was recorded along with the provider of the intervention, which could include EMS personnel, bystanders, or the patient themselves. Hypoglycemia was defined as a blood glucose level of ≤70 mg/dL, and severe hypoglycemia was defined as ≤50 mg/dL. Blood glucose levels were categorized as ≥70 mg/dL, 50–69 mg/dL, and <50 mg/dL for descriptive analysis.

Patient condition upon EMS arrival was assessed using information on level of consciousness, vital signs, and initial blood glucose levels when available. The level of consciousness was categorized as follows: Glasgow Coma Scale (GCS) scores of 15–14 corresponded to a one-digit Japan Coma Scale (JCS); GCS scores of 13–9 to a two-digit JCS; and GCS scores below 9 to a three-digit JCS [16]. From the ED records, data were collected on ED diagnoses, patient consciousness level at arrival, concurrent condition including hypothermia or infections, and treatments provided such as intravenous glucose administration. Clinical outcomes were categorized based on ED disposition, including hospitalization, discharge home, death in the ED, or other discharge destinations.

### 2.5. Outcome

The primary outcome of this study was to describe the occurrence and characteristics of hypoglycemia-related EMS transports. Specifically, the study analyzed the location and circumstances of hypoglycemia episodes, the time course of EMS response including the time from EMS call to EMS arrival and from EMS call to hospital arrival as well as patient condition at the time of EMS arrival and treatment details. Additionally, the study evaluated patient consciousness levels and clinical outcomes in the ED.

In the exploratory analysis, the study descriptively compared patient outcomes according to the presence or absence of prehospital interventions, such as glucose or glucagon administration, and patient outcomes. Furthermore, hospital interventions and their association with patient recovery and discharge outcomes were investigated.

### 2.6. Statistical Analysis

Categorical variables were described by number of cases and percentages, while continuous variables were described by mean, standard deviation, median, and interquartile range. Missing data were reported but not imputed, and a complete-case analysis was performed for the exploratory comparisons. No multivariable adjustment or casual modeling was conducted. Statistical analyses were performed using R version 4.2.2 (R Foundation for Statistical Computing, Vienna, Austria).

## 3. Results

### 3.1. Study Flowchart and Basic Characteristics

**Figure 1:**
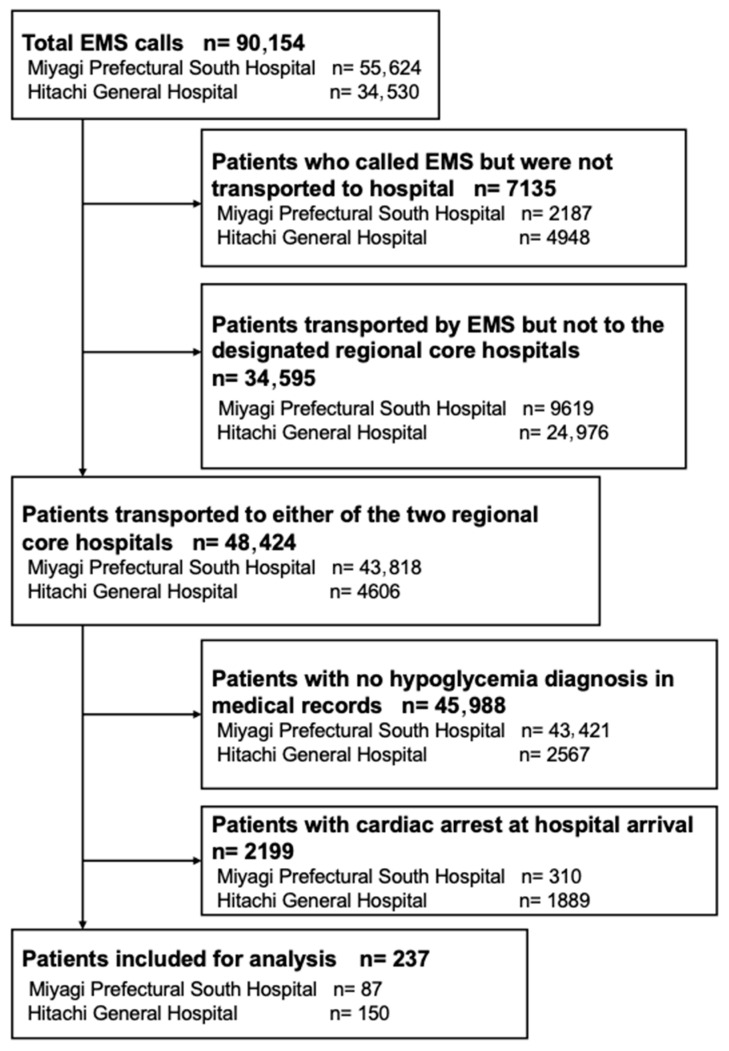
Study flow chart.

A total of 90,154 patients called EMS, and 237 patients were included in the final analysis (Figure 1). Patient characteristics are summarized in Table 1. The median age was 74 years (Q1: 53.4, Q3: 83.0). Of the included patients, 59.9% (142/237) were male. A history of type 1 diabetes was noted in 11.4% (27/237), and type 2 diabetes in 50.2% (119/237). Regular use of diabetes medication was reported in 41.8% (99/237) of cases, and among them, insulin was used in 58.6% (58/99).

### 3.2. Location and Time of Onset

**Figure 2:**
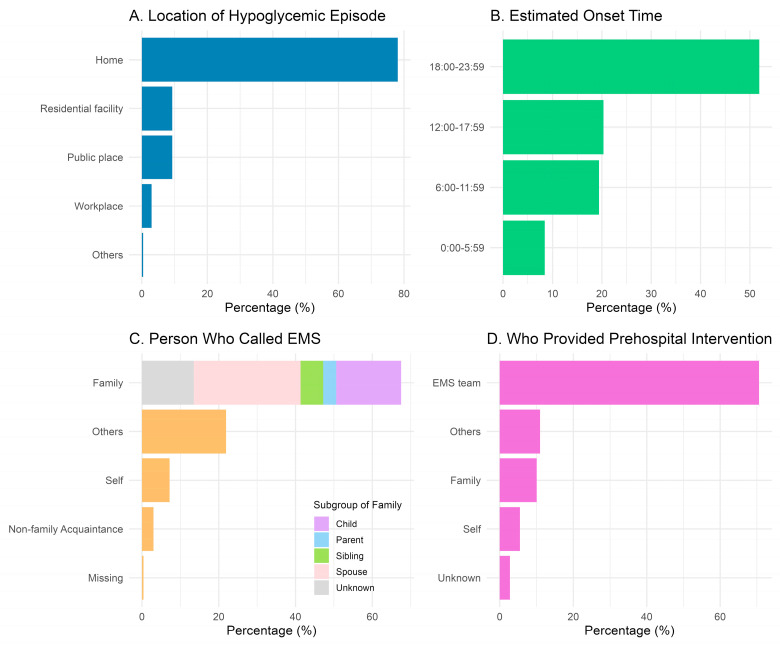
Characteristics of Emergency Medical Service Activations for Hypoglycemia. The figure summarizes the characteristics of Emergency Medical Service (EMS) activations for hypoglycemia, showing both the number and proportion of patients in each category. Panel (**A**) displays the location where the patient was found, with most cases occurring at home (78.1% (185/237)). Panel (**B**) presents the estimated time of symptom onset, with the highest frequency during the evening (51.9% (18:00–23:59; 123/237)). Panel (**C**) illustrates who identified and called EMS, showing that family members were the most common callers (67.5% (160/237)), with spouses (27.8% (66/237)) and children (16.9% (40/237) being the most frequent within this group. Panel (**D**) indicates who performed prehospital interventions for hypoglycemia, with EMS teams providing care in the majority of cases (68.8% (77/237)).

Hypoglycemic episodes occurred most frequently at home, accounting for 78.1% (185/237) of cases. Among the remaining cases, 9.3% (22/237) occurred in public places, 9.3% (22/237) were found in residential facilities, and 3.0% (7/237) occurred in workplaces. The estimated onset time was most commonly during the evening to nighttime hours, between 18:00 and 23:59, representing 51.9% (123/237) of cases. The most frequent dispatch complaint was consciousness disorder at 38.8% (92/237), while hypoglycemia itself accounted for 11.8% (28/237) (Table 1 and Figure 2).

### 3.3. EMS Caller and Bystander Interventions

Family members were the most commonly recorded as being involved at the time of the EMS call, accounting for 67.5% (160/237) of cases (Table 1), followed by the patient themselves at 7.2% (17/237). Among family members, spouses accounted for 41.3% (66/160), children for 25.0% (40/160), and siblings for 8.8% (14/160).

Prehospital interventions were performed in 47.3% (112/237) of cases. Among those who received interventions, self-management accounting for 5.4% (6/112), family-provided interventions accounted for 12.5% (14/112), while EMS teams were the most frequent providers, accounting for 68.8% (77/112) of cases.

Regarding the timing of interventions, approximately one third of patients received hypoglycemia treatment before EMS arrival (29.5%, 33/112). All patients treated by EMS was treated during transport, with 68.8% (77/112) taking place en route. As for the intervention methods, 65.2% (73/112) received intravenous glucose, and 20.5% (23/112) received oral glucose (Table 1 and Figure 2).

### 3.4. Patient Status at EMS Arrival

As shown in Table 2, at the time of EMS arrival, 16.0% (38/237) of patients were fully conscious. JCS scores were in the one-digit range for 27.8% (66/237), in the two-digit range for 26.1% (62/237), and in the three-digit range—indicating coma—for 21.1% (50/237). The initial blood glucose level measured upon EMS arrival had a median of 29.0 mg/dL (Q1: 20.0, Q3: 40.0), with 40.1% (95/237) of patients having a glucose level below 50 mg/dL (Table 2).

### 3.5. Timing and Management

The EMS glucose administration protocol was activated in 75.5% (179/237) of patients. Among those who received protocol-based treatment, the most common timing of intervention was during transport, accounting for 40.2% (72/179) of cases. The Patients who received glucose administered were 41.9% (75/179) of those treated under the protocol. The median time from EMS call to EMS arrival was 7.9 min (Q1: 5.0, Q3: 10.0), and the median time from EMS call to hospital arrival was 39.4 min (Q1: 28.0, Q3: 46.0) (Table 2).

### 3.6. Clinical Presentation After ED Arrival

Clinical presentations and related information at the ED are summarized in Table 3. At ED arrival, 14.3% (34/237) of patients were fully conscious, 20.7% (49/237) had a one-digit JCS score, 8.4% (20/237) had a two-digit score, and 8.9% (21/237) had a three-digit score. The initial blood glucose level measured after ED arrival had a median of 71.1 mg/dL (Q1: 22.1, Q3: 99.2). Among these patients, 33.8% (80/237) had a blood glucose level below 50 mg/dL; however, glucose data were missing in 40.5% (96/237) of cases (Table 3).

### 3.7. Treatment and Outcome

In the ED, 74.7% (177/237) of patients received treatment for hypoglycemia, all of whom were administered intravenous glucose. The most frequently observed concurrent condition was hypothermia, noted in 5.4% (13/237) of patients, followed by urinary tract infections in 2.5% (6/237), and disturbance of consciousness in 2.1% (5/237).

A total of 44.3% (105/237) of patients were hospitalized, all of whom were admitted to general wards. One patient (0.4%) died in the ED (Table 3).

**Figure 3:**
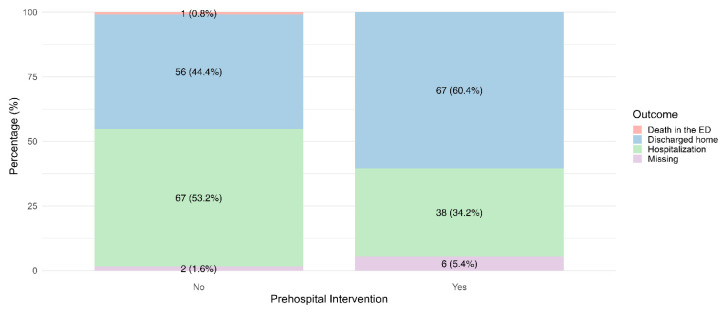
Distribution of Clinical Outcomes by Prehospital Intervention Status in Hypoglycemia. Among those without intervention, 53.2% (67/237) were hospitalized, 44.4% (56/237) were discharged home, 0.8% (1/237) died in the emergency department, and 1.6% (2/237) had missing outcome data. Among those who received intervention, 34.2% (38/237) were hospitalized, 60.4% (67/237) were go home, 0.0% died in the emergency department, and 5.4% (6/237) had missing outcome data.

**Figure 4:**
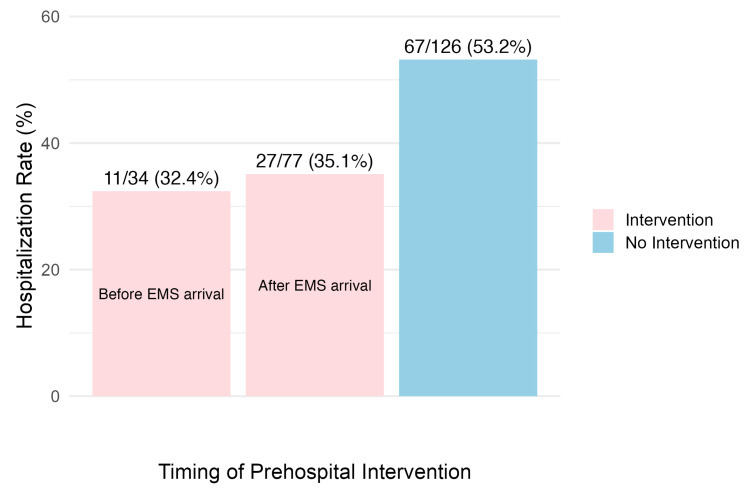
Hospitalization Rates by Timing of Prehospital Intervention. This figure illustrates the relationship between the timing of prehospital interventions for hypoglycemia and the proportion of patients who were subsequently hospitalized. Patients who received intervention before EMS arrival had the lowest hospitalization rate (32.4% (11/34)), followed by those who received intervention after EMS arrival (35.1% (27/77)). Patients who received no prehospital intervention had the highest hospitalization rate (53.2% (67/126)).

Additionally, the hospitalization rate was numerically lower among patients who received prehospital intervention (34.2%) compared to those who did not (53.2%) (Figure 3). In the graph comparing hospitalization rates by the timing of intervention, patients who received treatment before EMS arrival had the lowest hospitalization rate (Figure 4).

Facility-specific details are available in Supplementary Tables S1–S3.

## 4. Discussion

### 4.1. Key Findings and Strengths

This study provides a descriptive overview of the clinical characteristics and management of patients transported by EMS due to hypoglycemia in two regional EMS systems in Japan. Approximately 80% of episodes occurred at home during evening hours and were initiated by emergency calls made by family members, with nearly 40% of patients presenting with severe consciousness disturbance and blood glucose levels below 50 mg/dL. Prehospital interventions were frequently administered, primarily by EMS teams during transport, whereas only 30% of patients received any intervention before EMS arrival. Patients who received prehospital intervention showed a lower observed hospitalization rate in this descriptive comparison. However, because the analysis was retrospective and unadjusted, this difference should not be interpreted as evidence of a causal effect. These findings describe patterns of prehospital care in this cohort.

### 4.2. Interpretation

The findings of this study are consistent with previous reports, such as those based on EMS records of prehospital hypoglycemic events in the US [17,18] and an investigation into information-seeking behavior among caregivers of patients with type 2 diabetes in Europe [17,18]. These studies have shown that severe hypoglycemia requiring EMS activation often occurs at night or at home and is frequently identified by family members or caregivers.

In this study, 78% of episodes occurred at home and more than half occurred between 18:00 and 23:59, with family members accounting for 67.5% of EMS calls. These patterns reflect the influence of behavioral, pharmacologic, and social factors—such as mismatches between insulin action and food intake, diminished glucose monitoring during sleep, and caregiver presence on hypoglycemia recognition and escalation [19].

Notably, while family members made most EMS calls, their actual medical intervention was infrequent (12.5%). This gap suggests that although family members may detect hypoglycemia promptly, they often lack the knowledge, skills, or resources to initiate appropriate treatment. The vast majority of prehospital interventions were performed by EMS teams during transport, underscoring the critical role of EMS protocols and training in mitigating progression to severe outcomes.

More than 20% of patients were in a coma (JCS 3-digit) at EMS arrival, and 40% had glucose levels below 50 mg/dL, indicating that many episodes had already progressed to a critical stage by the time of professional contact. The lower hospitalization rate observed among patients who received prehospital intervention may reflect differences in clinical severity or other unmeasured factors rather than a direct effect of the intervention itself [20,21]. Given the descriptive and unadjusted nature of this analysis, no causal inference can be made.

### 4.3. Clinical and Research Implication

Our findings provide descriptive information on the circumstances surrounding hypoglycemia-related EMS transports, including the role of family members in recognizing events and initiating EMS calls. Because this study did not assess caregiver knowledge, barriers to intervention, or outcomes following educational initiatives, no conclusions can be drawn regarding the effectiveness of caregiver education or related interventions. Future studies are needed to investigate barriers to family-initiated interventions and to evaluate educational or technological approaches (e.g., automated alerts or home glucose rescue kits) in this context. Community-based strategies, such as public campaigns and structured caregiver training programs, have already been implemented in several Western countries [22]. However, the applicability of such approaches to regional healthcare systems in Japan remains uncertain and warrants further investigation.

## 5. Limitations

This study has several limitations. First, the study was conducted in only two regions of Japan, both with centralized EMS systems. Because the study population represents two regional EMS catchment areas rather than the entire Japanese population, the findings should be interpreted as regional observations rather than nationally representative estimates, which may limit the generalizability of the findings to other geographic areas with differing healthcare infrastructure or patient demographics. Second, patients who were not transported to hospitals were excluded from the analysis. Among these were cases in which EMS was activated but the patients were not ultimately transported. This group likely included individuals who recovered after receiving prehospital treatment either by bystander or EMS personnel at the scene. Excluding such cases may have led to an underestimation of the effect of prehospital interventions and introduced selection bias. Therefore, the study population may be biased toward patients with more severe or persistent hypoglycemia who ultimately required hospital transport. Accordingly, the present study should be interpreted as a description of hypoglycemia-related EMS transports rather than the full epidemiology of hypoglycemic events within the community. In addition, detailed information on underlying causes of hypoglycemia, such as sepsis, alcohol use, or malnutrition, was not available in the dataset. Therefore, hypoglycemia was not etiologically classified. It is also possible that a small number of cases involving glucose administration reflected precautionary treatment rather than confirmed hypoglycemia. Medication data were derived from structured electronic medication fields, and the completeness and accuracy of documentation could not be independently verified. Therefore, absence of recorded medication may reflect either true non-use or incomplete data entry. Additionally, recently approved agents, such as GIP/GLP-1 receptor agonists introduced late in the study period, may be underrepresented. Third, there were substantial missing data for key clinical variables, such as blood glucose levels and JCS, both at EMS arrival and at hospital arrival. We recognize that the degree of missingness represents an important limitation of the present study and may reduce the interpretability and robustness of some findings. These missing data may have limited the ability to accurately assess and compare patient severity, thus potentially affecting the reliability of the results. One possible reason for this missingness is routine clinical documentation practice. In many cases, patients transported for hypoglycemia had already regained consciousness by the time of ED arrival, and detailed consciousness scales such as the GCS or JCS were not always recorded in the medical chart. Additionally, blood glucose is often measured using point-of-care testing devices rather than formal laboratory tests, and these values are not always captured in structured electronic medical record fields, which limited their availability in the dataset used for this analysis. This pattern of missingness may have introduced information bias, and depending on whether the likelihood of documentation differed by patient condition, differential misclassification cannot be ruled out. More rigorous evaluation of these clinical variables would likely require prospectively collected data with standardized documentation protocols. Fourth, patients who were in cardiac arrest upon EMS arrival were excluded from this study. Severe hypoglycemia has been reported to impose cardiovascular stress through mechanisms such as QT interval prolongation and potentially fatal arrhythmias [15]. However, given the difficulty in determining whether cardiac arrest was caused by hypoglycemia, such cases were excluded to avoid including patients with heterogeneous etiologies. As a result, whether severe hypoglycemia can directly induce cardiac arrest remains unclear.

Finally, due to the retrospective, observational design of the study, no causal inferences can be made between prehospital interventions and clinical outcomes, nor were we able to evaluate long-term outcomes such as readmissions or post-discharge complications.

## 6. Conclusions

This study described the characteristics and situational patterns of hypoglycemia-related EMS transports in two regional EMS systems in Japan. Although most episodes were discovered by family members, actual prehospital intervention by bystanders was infrequent. Instead, EMS teams provided the majority of care before hospital arrival. Notably, patients who received intervention at an earlier stage especially before EMS arrival showed lower observed hospitalization rates in this descriptive comparison. These findings describe pattern related to EMS activation in this cohort. Further studies are needed to better understand factors influencing prehospital responses to hypoglycemia.

## Figures and Tables

**Table 1:** Characteristics and circumstances of patients with severe hypoglycemia in the prehospital setting.

Variables	Total N = (237)
**Age (year), median, IQR**	74.0 (53.4, 83)
**Male**	142 (59.9%)
**BMI (kg/m^2^)**	20.6 (17.3, 24.3)
Missing	46 (23.7%)
**Past medical history**	
Type 1 diabetes	27 (11.4%)
Type 2 diabetes	119 (50.2%)
Diabetes other	9 (3.8%)
Unspecified (e.g., only “diabetes”)	82 (54.7%)
**Regular use of diabetes medication**	105 (44.3%)
**Details of medication** **(*n* = 105)**	
Insulin	65 (61.9%)
Biguanide class	19 (18.1%)
Thiazolidinedione class	8 (7.6%)
DPP-4 inhibitors	44 (41.9%)
Sulfonylurea class	19 (18.1%)
Glinide class	5 (4.8%)
Alpha-glucosidase inhibitors	22 (21.0%)
SGLT2 inhibitors	28 (26.7%)
GLP-1 receptor agonist	4 (3.8%)
**Location**	
Home	185 (78.1%)
Public place (*n* = 22)	22 (9.3%)
- Public road/street/park	9 (40.9%)
- Short-term care facility	1 (4.5%)
- Lodging facility	1 (4.5%)
- Gaming/entertainment venue	1 (4.5%)
- Farm/agricultural facility	1 (4.5%)
- Retail store	1 (4.5%)
- Supermarket	1 (4.5%)
- Unknown/unspecified	7 (31.8%)
Residential facility	22 (9.3%)
Workplace	7 (3.0%)
Others/unspecified	1 (0.4%)
**Estimated onset time**	
0:00–5:59	20 (8.4%)
6:00–11:59	46 (19.4%)
12:00–17:59	48 (20.3%)
18:00–23:59	123 (51.9%)
**Dispatch complaint**	
Hypoglycemia	28 (11.8%)
Consciousness disorder	92 (38.8%)
Others	42 (17.7%)
Missing	75 (31.6%)
**Person who discovered the patient or was involved in contacting EMS**	
Self	17 (7.2%)
Family (*n* = 160)	160 (67.5%)
- Spouse	66 (41.3%)
- Parent	8 (5.0%)
- Sibling	14 (8.8%)
- Child	40 (25.0%)
- Unknown	32 (20.0%)
Non-family Acquaintance (e.g., relative, acquaintance)	7 (3.0%)
Others (e.g., coworker, care facility staff, daycare service, taxi driver)	52 (21.9%)
Missing	1 (0.4%)
**Prehospital Intervention for hypoglycemia**	112 (47.3%)
**Who**	
Self	6 (5.4%)
Family	14 (12.5%)
- Spouse	6 (5.4%)
- Child	5 (4.5%)
- Unknown	3 (2.7%)
EMS team	77 (68.8%)
Others	12 (10.7%)
Missing/unspecified	3 (2.7%)
**When**	
Before EMS arrival	33 (29.5%)
During transport	77 (68.8%)
Missing	2 (1.8%)
**What**	
Oral glucose intake	23 (20.5%)
Intravenous glucose administration	73 (65.2%)
Candy	7 (6.3%)
Juice	1 (0.9%)
Other meals	4 (3.6%)
Missing	4 (3.6%)

Categorical variables: *n* (%). Continuous variables: median, Q1 and Q3. BMI: Body Mass Index, EMS: Emergency Medical Services.

**Table 2:** Prehospital assessment, intervention, and timeline.

Variables	Total N = (237)
**Vital signs on EMS arrival**	
Japan Coma Scale (JCS)	
Alert	38 (16.0%)
One-digit range (GCS 15–14)	66 (27.8%)
Two-digit range (GCS 13–9)	62 (26.1%)
Three-digit range (GCS < 9)	50 (21.1%)
Missing of JCS	21 (8.9%)
Body temperature (BT) (°C)	36.1 (35, 36.5)
Missing of BT	33 (13.9%)
Heart rate (HR) (/min)	83 (66, 96)
Missing of HR	23 (9.7%)
Systolic blood pressure (SBP) (mmHg)	150 (111.3, 175)
Missing of SBP	45 (19.0%)
Diastolic blood pressure (DBP) (mmHg)	84 (62, 99.2)
Missing of DBP	48 (20.3%)
Respiratory rate (RR) (/min)	20 (18, 24)
Missing of RR	31 (13.1%)
SpO_2_ (%)	97 (92.3, 99)
Missing of SpO_2_	34 (14.3%)
**Initial blood sugar level on EMS arrival (mg/dL)**	29 (20, 40)
≥70	4 (1.7%)
50–69	8 (3.4%)
<50	95 (40.1%)
Missing	130 (54.9%)
**Activation of glucose administration protocol by emergency team**	179 (75.5%)
**When**	
From EMS arrival to departure	5 (2.8%)
During transport	72 (40.2%)
Missing	102 (57.0%)
**What**	
Glucose administration	75 (41.9%)
Missing	162 (58.1%)
**Timeline regarding hypoglycemia (min)**	
From EMS call to EMS arrival	7 (5, 10)
From EMS call to hospital arrival	38 (32, 46)
**Time from emergency call to arrival on scene, min**	
On-site duration (EMS arrival–EMS departure)	17 (11, 23)
Total consultation time	4 (2.3, 9.5)
Total transport time (EMS call to hospital arrival)	37 (27, 44)

Categorical variables: *n* (%). Continuous variables: median, Q1 and Q3. GCS: Glasgow Coma Scale, EMS: Emergency Medical Services.

**Table 3:** Details of management of severe hypoglycemia in the emergency department.

Variables	Total N = (237)
**Vital signs at the ED**	
Japan Coma Scale (JCS)	
Alert	34 (14.3%)
One-digit range (GCS 15–14)	49 (20.7%)
Two-digit range (GCS 13–9)	20 (8.4%)
Three-digit range (GCS < 9)	21 (8.9%)
Missing of JCS	113 (47.7%)
Body temperature (BT) (°C)	35.8 (33.7, 36.4)
Missing of BT	73 (30.8%)
Heart rate (HR) (/min)	81 (66, 96)
Missing of HR	69 (29.1%)
Systolic blood pressure (SBP) (mmHg)	142 (100, 171.8)
Missing of SBP	70 (29.5%)
Diastolic blood pressure (DBP) (mmHg)	81 (58.2, 94)
Missing of DBP	70 (29.5%)
Respiratory rate (RR) (/min)	20 (15, 23)
Missing of RR	75 (31.6%)
SpO_2_ (%)	98 (95, 99.8)
Missing of SpO_2_	83 (35.0%)
**Chief complaint at ED**	
Disturbance of consciousness	98 (41.4%)
Seizure	11 (4.6%)
Others	53 (22.4%)
Missing	75 (31.6%)
**Initial blood sugar level at the ED (mg/dL)**	44.5 (22.1, 99.2)
≥70	45 (19.0%)
50–69	16 (6.8%)
<50	80 (33.8%)
Missing	96 (40.5%)
**Intervention for hypoglycemia in the ED**	177 (74.7%)
Glucose administration	177 (100.0%)
**Primary diagnosis at the ED**	
Hypoglycemia	150 (63.3%)
Hypoglycemic attack	56 (23.6%)
Hypoglycemic encephalopathy	2 (0.8%)
**Concurrent conditions**	
Hypothermia	13 (5.4%)
Urinary tract infection	6 (2.5%)
Disturbance of consciousness	5 (2.1%)
Heart failure	4 (1.7%)
Hypokalemia	4 (1.7%)
**Prognosis**	
Discharged home	123 (51.9%)
Hospitalization	105 (44.3%)
Death in the ED	1 (0.4%)
Missing	8 (3.4%)
**Admission ward**	
General ward	105 (100.0%)

Categorical variables: *n* (%). Continuous variables: median, Q1 and Q3. ED: Emergency Department, GCS: Glasgow Coma Scale.

## Data Availability

The original contributions presented in this study are included in the article/Supplementary Material. Further inquiries can be directed to the corresponding author.

## Supplementary Materials

The following supporting information can be downloaded at https://www.mdpi.com/article/10.3390/jcm15072746/s1: Supplementary Table S1. Facility-specific characteristics and circumstances of patients with severe hypoglycemia in the prehospital setting; Supplementary Table S2. Facility-specific prehospital assessment, intervention, and timeline; Supplementary Table S3. Facility-specific details of management of severe hypoglycemia in the emergency department; Supplementary Table S4. Missing data-specific characteristics and circumstances of patients with severe hypoglycemia in the prehospital setting; Supplementary Table S5. Missing data-specific prehospital assessment, intervention, and timeline; Supplementary Table S6. Missing data-specific details of management of severe hypoglycemia in the emergency department; Supplementary Figure. Hospital location in Japan.

## References

[1] Amiel S.A. (2021). The consequences of hypoglycaemia. Diabetologia.

[2] Lacy M.E., Gilsanz P., Eng C., Beeri M.S., Karter A.J., Whitmer R.A. (2020). Severe Hypoglycemia and Cognitive Function in Older Adults With Type 1 Diabetes: The Study of Longevity in Diabetes (SOLID). Diabetes Care.

[3] Cryer P.E. (2012). Severe hypoglycemia predicts mortality in diabetes. Diabetes Care.

[4] Zoungas S., Patel A., Chalmers J., de Galan B.E., Li Q., Billot L., Woodward M., Ninomiya T., Neal B., MacMahon S. (2010). Severe hypoglycemia and risks of vascular events and death. N. Engl. J. Med..

[5] Heller S.R., Peyrot M., Oates S.K., Taylor A.D. (2020). Hypoglycemia in patient with type 2 diabetes treated with insulin: It can happen. BMJ Open Diabetes Res. Care.

[6] Geller A.I., Shehab N., Lovegrove M.C., Kegler S.R., Weidenbach K.N., Ryan G.J., Budnitz D.S. (2014). National estimates of insulin-related hypoglycemia and errors leading to emergency department visits and hospitalizations. JAMA Intern. Med..

[7] McCoy R.G., Van Houten H.K., Ziegenfuss J.Y., Shah N.D., Wermers R.A., Smith S.A. (2012). Increased mortality of patients with diabetes reporting severe hypoglycemia. Diabetes Care.

[8] Brackenridge A., Wallbank H., Lawrenson R.A., Russell-Jones D. (2006). Emergency management of diabetes and hypoglycaemia. Emerg. Med. J..

[9] Mattishent K., Loke Y.K. (2016). Bi-directional interaction between hypoglycaemia and cognitive impairment in elderly patients treated with glucose-lowering agents: A systematic review and meta-analysis. Diabetes Obes Metab.

[10] Statistics Bureau of Japan 2020 Population Census of Japan (Kokusei Chosa). https://www.stat.go.jp/data/kokusei/2020/kekka.html.

[11] Government I.P. Statistical Profile of Hitachi City. https://www.pref.ibaraki.jp/kikaku/tokei/fukyu/tokei/sugata/local/hitachi.html.

[12] Saito A., Osawa I., Shibata J., Sonoo T., Nakamura K., Goto T. (2023). The prognostic utility of prehospital qSOFA in addition to emergency department qSOFA for sepsis in patients with suspected infection: A retrospective cohort study. PLoS ONE.

[13] Okada Y., Okada A., Ito H., Sonoo T., Goto T. (2022). External validation of the POP score for predicting obstetric and gynecological diseases in the emergency department. Am. J. Emerg. Med..

[14] Goto T., Hara K., Hashimoto K., Soeno S., Shirakawa T., Sonoo T., Nakamura K. (2020). Validation of chief complaints, medical history, medications, and physician diagnoses structured with an integrated emergency department information system in Japan: The Next Stage ER system. Acute Med. Surg..

[15] Tsujimoto T., Yamamoto-Honda R., Kajio H., Kishimoto M., Noto H., Hachiya R., Kimura A., Kakei M., Noda M. (2014). Vital signs, QT prolongation, and newly diagnosed cardiovascular disease during severe hypoglycemia in type 1 and type 2 diabetic patients. Diabetes Care.

[16] Nakajima M., Okada Y., Sonoo T., Goto T. (2023). Development and Validation of a Novel Method for Converting the Japan Coma Scale to Glasgow Coma Scale. J. Epidemiol..

[17] Woodburn E.V., Rostykus P.S. (2019). Prehospital Management of Hypoglycemic Emergencies: Evidence-Based Review for Collegiate-Based Emergency Medical Services. J. Coll. Emerg. Med. Serv..

[18] Reifegerste D., Hartleib S. (2016). Hypoglycemia-related information seeking among informal caregivers of type 2 diabetes patients: Implications for health education. J. Clin. Transl. Endocrinol..

[19] Naha S., Gardner M.J., Khangura D., Kurukulasuriya L.R., Sowers J.R., Feingold K.R., Adler R.A., Ahmed S.F., Anawalt B., Blackman M.R., Chrousos G., Corpas E., de Herder W.W., Dhatariya K., Dungan K. (2021). Hypoglycemia in Diabetes. Endotext.

[20] O’Connor L., Kue R.C., O’Connor M.J. (2019). Characteristics of Patients with Recurrent Emergency Medical Services Utilization for Symptomatic Hypoglycemia in an Urban Setting. Prehosp Emerg. Care.

[21] Jeung S., Kang S.M., Seo Y., Yu H., Baek C.H., Kim H., Yang W.S., Park S.K. (2018). A Case Series of Asymptomatic Hemodialysis Catheter-Related Right Atrial Thrombi That Are Incidentally Detected Prior to Kidney Transplantation. Transplant. Proc..

[22] O’Donnell H.K., Vigers T., Johnson S.B., Pyle L., Gonder-Fredrick L., Hendrieckx C., Driscoll K.A. (2022). Bring Blood Glucose Down! An intervention to reduce fear of hypoglycemia in caregivers of adolescents with type 1 diabetes: Study design and participant characteristics. Contemp. Clin. Trials.

