# Supplementary material for: Hypoglycemic Events Focusing on Situational Factors, Bystander Identification, and Prehospital Management
Source: J Clin Med. 2026 Apr 5;15(7):2746. doi: 10.3390/jcm15072746 (PMC13073431; doi:10.3390/jcm15072746)
Supplement: Supplementary file 1 [file jcm-15-02746-s001.zip › Hypoglycemia_Supplementary_File.pdf]

Supplemental Materials

Table S1. Facility-specific characteristics and circumstances of patients with severe hypoglycemia in the prehospital setting

Variables	Total N= (237)				Miyagi Prefectural South Hospital N= (87)				Hitachi General Hospital N= (150)			
	n (%)	Mean (SD)	min, max	Median (Q1, Q3)	n (%)	Mean (SD)	min, max	Median (Q1, Q3)	n (%)	Mean (SD)	min, max	Median (Q1, Q3)
**Age (year)**		69.7 (18.6)	1, 104	74 (53.4, 83.0)		66.4 (19.0)	17, 100	71 (45.4, 81.0)		71.6 (18.2)	1, 104	76.5 (57.4, 84.0)
**Male**	142 (59.9%)				55 (63.2%)				87 (58.0%)			
**BMI (kg/m^2)**		21.4 (4.5)	10.3, 46.3	20.6 (17.3, 24.3)		21.8 (4.4)	12.7, 27.7	20.9 (16.8, 25.5)		21.2 (4.6)	10.3, 46.3	20.3 (17.4, 23.3)
Missing	46 (23.7%)				20 (23.0%)				30 (17.3%)			
**Past medical history**												
Type 1 diabetes	27 (11.4%)				2 (2.3%)				25 (16.7%)			
Type 2 diabetes	119 (50.2%)				43 (49.4%)				76 (50.7%)			
Diabetes other	9 (3.8%)								9 (6.0%)			
Diabetes associated with pancreatic cancer	7 (3.0%)								7 (4.7%)			
Diabetes secondary to chronic pancreatitis	1 (0.4%)								1 (0.7%)			
Mitochondrial disorder	1 (0.4%)								1 (0.7%)			
Unspecified (e.g., only "diabetes")	82 (54.7%)				42 (48.3%)				40 (26.7%)			
**Regular use of diabetes medication**	105 (44.3%)				33 (37.9%)				72 (48.0%)			
**Details of medication**	(n=105)				(n=33)				(n=72)			
Insulin	65 (61.9%)				27 (81.8%)				38 (52.8%)			

Biguanide class	19 (18.1%)	6 (18.2%)	13 (18.1 %)
Thiazolidinedione class	8 (7.6%)	2 (6.1%)	6 (8.3%)
DPP-4 inhibitors	44 (41.9%)	10 (30.3%)	34 (47.2%)
Sulfonylurea class	19 (18.1%)	2 (6.1%)	17 (23.6%)
Glinide class	5 (4.8%)	0 (0.0%)	5 (6.9%)
Alpha-glucosidase inhibitors	22 (21.0%)	6 (18.2%)	16 (22.2%)
SGLT2 inhibitors	28 (26.7%)	13 (39.4%)	15 (20.8%)
GLP-1 receptor agonist	4 (3.8%)	0 (0.0%)	4 (5.6%)
**Location**			
Home	185 (78.1%)	70 (80.5%)	115 (76.7%)
Public place (n=22)	22 (9.3%)	7 (8.0%)	15 (10.0%)
Public road/street/park	9 (40.9%)	0 (0%)	9 (60.0%)
Short-term care facility	1 (4.5%)	0 (0%)	1 (6.7%)
Lodging facility	1 (4.5%)	0 (0%)	1 (6.7%)
Gaming/entertainment venue	1 (4.5%)	0 (0%)	1 (6.7%)
Farm/agricultural facility	1 (4.5%)	0 (0%)	1 (6.7%)
Retail store	1 (4.5%)	0 (0%)	1 (6.7%)
Supermarket	1 (4.5%)	0 (0%)	1 (6.7%)
Unknown/unspecified	7 (31.8%)	7 (100%)	0 (0%)
Residential facility	22 (9.3%)	7 (8.0%)	15 (10.0%)
Workplace	7 (3.0%)	2 (2.3%)	5 (3.3%)
Others/unspecified	1 (0.4%)	1 (1.1%)	0 (0%)
**Estimated onset time**			
0:00-5:59	20 (8.4%)	10 (11.5%)	10 (6.7%)
6:00-11:59	46 (19.4%)	25 (28.7%)	21 (14.0%)

12:00-17:59	48 (20.3%)	26 (29.9%)	22 (14.7%)
18:00-23:59	123 (51.9%)	26 (29.9%)	97 (64.7%)
**Dispatch complaint**			
Hypoglycemia	28 (11.8%)	12 (13.8%)	16 (10.7%)
Consciousness disorder	92 (38.8%)	27 (31.0%)	65 (43.3%)
Others	42 (17.7%)	7 (8.0%)	35 (23.3%)
Missing	75 (31.6%)	41 (47.1%)	34 (22.7%)
**Person who discovered the patient or was involved in contacting EMS**			
Self	17 (7.2%)	6 (6.9%)	11 (7.3%)
Family (n=160)	160 (67.5%)	61 (70.1%)	99 (66.0%)
Spouse	66 (27.8%)	15 (17.2%)	51 (34.0%)
Parent	8 (3.4%)	4 (4.6%)	4 (2.7%)
Sibling	14 (5.9%)	9 (10.3%)	5 (3.3%)
Child	40 (16.9%)	10 (11.5%)	30 (20.0%)
Unknown	32 (13.5%)	23 (26.4%)	9 (6.0%)
Non-family Acquaintance (e.g., relative、acquaintance)	7 (3.0%)	4 (4.6%)	3 (2.0%)
Others （e.g., coworker, care facility staff, daycare service, taxi driver）	52 (21.9%)	16 (18.4%)	36 (24.0%)
Missing	1 (0.4%)	0 (0.0%)	1 (0.7%)
**Prehospital Intervention for hypoglycemia**	112 (47.3%)	42 (48.3%)	70 (46.7%)
**Who**			
Self	6 (5.4%)	0 (0.0%)	6 (8.6%)
Family	14 (12.5%)	3 (7.1%)	11 (15.7%)
Spouse	6 (5.4%)	0 (0.0%)	6 (8.6%)
Child	5 (4.5%)	0 (0.0%)	5 (7.1%)

Unknown	3 (2.7%)	3 (7.1%)	0 (0.0%)
Non-family Acquaintance	0 (0.0%)	0 (0.0%)	0 (0.0%)
EMS team	77 (68.8%)	34 (81.0%)	43 (61.4%)
Others	12 (10.7%)	3 (7.1%)	9 (12.9%)
Missing/unspecified	3 (2.7%)	2 (4.8%)	1 (1.4%)
**When**			
Before EMS arrival	33 (29.5%)	9 (21.4%)	24 (34.3%)
During transport	77 (68.8%)	31 (73.8%)	46 (65.7%)
Missing	2 (1.8%)	2 (4.8%)	0 (0.0%)
**What**			
Oral glucose intake	23 (20.5%)	4 (9.5%)	19 (27.1%)
Intravenous glucose administration	73 (65.2%)	32 (76.2%)	41 (58.6%)
Candy	7 (6.3%)	2 (4.8%)	5 (7.1%)
Juice	1 (0.9%)	0 (0.0%)	1 (1.4%)
Other meals	4 (3.6%)	0 (0.0%)	4 (5.7%)
Missing	4 (3.6%)	4 (9.5%)	0 (0.0%)

Categorical variables: n (%). Continuous variables: mean, standard deviation (SD) ,min, max, median, Q1 and Q3.

BMI: Body Mass Index,

EMS: Emergency Medical Services.

Table S2. Facility-specific prehospital assessment, intervention, and timeline

**Variables**	**Total N= (237)**				**Miyagi Prefectural South Hospital N= (87)**				**Hitachi General Hospital N= (150)**			
	n (%)	Mean (SD)	min, max	Median (Q1, Q3)	n (%)	Mean (SD)	min, max	Median (Q1, Q3)	n (%)	Mean (SD)	min, max	Median (Q1, Q3)

**Vital signs on EMS arrival**												
**Japan Coma Scale (JCS)**												
Alert	38 (16.0%)				9 (10.3%)				29 (19.3%)			
I-1	21 (8.9%)				4 (4.6%)				17 (11.3%)			
I-2	10 (4.2%)				3 (3.4%)				7 (4.7%)			
I-3	35 (14.8%)				11 (12.6%)				24 (16.0%)			
II-10	37 (15.6%)				18 (20.7%)				19 (12.7%)			
II-20	12 (5.1%)				4 (4.6%)				8 (5.3%)			
II-30	13 (5.5%)				3 (3.5%)				10 (6.7%)			
III-100	17 (7.2%)				4 (4.6%)				13 (8.7%)			
III-200	11 (4.6%)				6 (6.9%)				5 (3.3%)			
III-300	22 (9.3%)				5 (5.8%)				17 (11.3%)			
Missing of JCS	21 (8.9%)				20 (23.0%)				1 (0.7%)			
**Body temperature (BT) (℃)**		35.9 (1.4)	31.2, 40.1	36.1 (35.0, 36.5)		35.6 (1.5)	31.2, 40.1	35.8 (34.2, 36.3)		36.0 (1.3)	31.3, 39.3	36.2 (35.3, 36.6)
Missing of BT	33 (13.9%)				24 (27.6%)				9 (6.0%)			
**Heart rate (HR) (/min)**		86.3 (21.1)	20, 180	83 (66.0, 96.0)		84.4 (17.6)	46, 148	82 (69.0, 94.8)		87.2 (22.4)	20, 180	84 (66.0, 98.5)
Missing of HR	23 (9.7%)				21 (24.1%)				2 (1.3%)			
**Systolic blood pressure (SBP) (mmHg)**		149.8 (37.6)	50, 268	150 (111.3, 175.0)		152.1 (38.0)	89, 268	151 (109.2, 175.5)		149.5 (36.8)	50, 235	150 (114.1, 175.0)
Missing of SBP	45 (19.0%)				24 (27.6%)				23 (15.3%)			
**Diastolic blood pressure (DBP) (mmHg)**		86.0 (25.1)	37, 190	84 (62.0, 99.2)		81.6 (22.1)	11, 140	84 (61.0, 95.5)		87.7 (27.1)	37, 190	84 (62.8, 100.8)
Missing of DBP	48 (20.3%)				24 (27.6%)				24 (16.0%)			
**Respiratory rate (RR) (/min)**		20.9 (4.5)	10, 45	20 (18.0, 24.0)		19.4 (4.6)	12, 45	18 (16.4, 20.0)		21.4 (4.3)	10, 36	20 (18.0, 24.0)
Missing of RR	31 (13.1%)				30 (34.5%)				1 (0.7%)			

**SpO_2 (%)**		95.6 (5.7)	66, 100	97 (92.3, 99.0)		96.8 (2.9)	85, 100	97 (94.6, 99.0)		95.0 (6.5)	66, 100	98 (90.0, 99.0)
Missing of SpO_2	34 (14.3%)				22 (25.3%)				12 (8.0%)			
**Initial blood sugar level on EMS arrival (mg/dl)**		37.0 (37.4)	10, 288	29 (20.0, 40.0)		37.5 (35.9)	10, 288	32 (21.0, 40.0)		36.5 (39.4)	14, 285	26 (20.0, 39.0)
≥ 70	4 (1.7%)				1 (1.2%)				3 (2.0%)			
50-69	8 (3.4%)				5 (5.8%)				3 (2.0%)			
< 50	95 (40.1%)				50 (57.5%)				45 (30.0%)			
Missing	130 (54.9%)				31 (35.6%)				99 (66.0%)			
**Activation of glucose administration protocol by emergency team**	179 (75.5%)				70 (80.5%)				109 (72.7%)			
**When**												
From EMS arrival to departure	5 (2.8%)				3 (4.3%)				2 (1.8%)			
During transport	72 (40.2%)				31 (44.3%)				41 (37.6%)			
Missing	102 (57.0%)				36 (51.4%)				66 (60.6%)			
**What**												
Glucose administration	75 (41.9%)				32 (45.7%)				43 (39.4%)			
Missing	162 (58.1%)				55 (54.3%)				107 (60.6%)			
**Timeline regarding hypoglycemia (min)**												
From EMS call to EMS arrival		7.9 (3.4)	1, 19	7 (5.0, 10.0)		10.4 (3.4)	4, 19	10 (7.0,13.0)		6.5 (2.4)	1, 15	6 (4.0, 7.0)
From EMS call to hospital arrival		39.4 (11.1)	18, 74	38 (32.0, 46.0)		44.1 (12.4)	23, 74	42 (31.8, 51.5)		36.7 (9.1)	18, 70	37 (27.0, 41.0)
**Time from emergency call to arrival on scene, min**												
On-site duration (EMS arrival – EMS departure)		18.4 (7.8)	0, 57	17 (11.0, 23.0)		19.3 (8.2)	0, 57	18 (12.9, 23.0)		17.9 (7.5)	6, 43	16.5 (10.0, 22.0)

Total consultation time		15.0 (44.0)	0, 355	4 (2.0, 9.5)		21.4 (63.1)	0, 355	3.5 (2.0,10.2)		9.5 (13.1)	0, 57	4 (2.1, 8.0)
Total transport time (EMS call to hospital arrival)		38.2 (11.3)	17, 74	37 (27.0, 44.0)		44.1 (12.4)	23, 74	42 (31.8, 51.5)		34.7 (9.0)	17, 68	35 (25.0, 40.0)

Categorical variables: n (%). Continuous variables: mean, standard deviation (SD) ,min, max, median, Q1 and Q3.

GCS: Glasgow Coma Scale, EMS: Emergency Medical Services.

Table S3. Facility-specific details of management of severe hypoglycemia in the emergency department

**Variables**	**Total N= (237)**				**Miyagi Prefectural South Hospital N= (87)**				**Hitachi General Hospital N= (150)**			
	n (%)	Mean (SD)	min, max	Median (Q1, Q3)	n (%)	Mean (SD)	min, max	Median (Q1, Q3)	n (%)	Mean (SD)	min, max	Median (Q1, Q3)
**Vital signs at the ED**												
Japan Coma Scale (JCS)												
Alert	34 (14.3%)				10 (11.5%)				24 (16.0%)			
I-1	24 (10.1%)				16 (18.4%)				8 (5.3%)			
I-2	10 (4.2%)				7 (8.0%)				3 (2.0%)			
I-3	15 (6.3%)				9 (10.3%)				6 (4.0%)			
II-10	8 (3.4%)				2 (2.3%)				6 (4.0%)			
II-20	8 (3.4%)				3 (3.4%)				5 (3.3%)			
II-30	4 (1.7%)				1 (1.1%)				3 (2.0%)			
III-100	5 (2.1%)				1 (1.1%)				4 (2.7%)			
III-200	7 (3%)				7 (8.0%)				0 (0.0%)			
III-300	9 (3.8%)				4 (4.6%)				5 (3.3%)			
Missing of JCS	113 (47.7%)				27 (31.0%)				86 (57.3%)			
Body temperature (BT) (℃)		35.3 (1.7)	28, 38.9	35.8 (33.7, 36.4)		35.2 (1.9)	28, 38.9	35.5 (34.0, 36.3)		35.3 (1.6)	31.1, 38.3	35.8 (33.7, 36.4)
Missing of BT	73 (30.8%)				31 (35.6%)				42 (28.0%)			

Heart rate (HR) (/min)		83.4 (19.3)	20, 166	81 (66.0, 96.0)		80.8 (16.4)	40, 134	79 (66.0, 91.2)		84.8 (20.6)	20, 166	83 (65.0, 96.0)
Missing of HR	69 (29.1%)				27 (31.0%)				42 (28.0%)			
Systolic blood pressure (SBP) (mmHg)		143.9 (37.5)	54, 250	142 (100.0, 171.8)		148.7 (38.3)	76, 250	144 (114.9, 176.0)		141.3 (37.0)	54, 229	142 (99.0, 169.5)
Missing of SBP	70 (29.5%)				28 (32.2%)				42 (28.0%)			
Diastolic blood pressure (DBP) (mmHg)		81 (20.8)	41, 166	81 (58.2, 94.0)		82.4 (20.2)	43, 166	83 (64.8, 92.0)		80.3 (21.2)	41, 135	79.5 (57.4, 95.8)
Missing of DBP	70 (29.5%)				28 (32.2%)				42 (28.0%)			
Respiratory rate (RR) (/min)		19.7 (5.1)	11, 40	20 (15.0, 23.0)		18.8 (4.1)	12, 30	18 (14.4, 21.0)		20.3 (5.5)	11, 40	20 (15.0, 24.0)
Missing of RR	75 (31.6%)				30 (34.5%)				45 (30.0%)			
SpO_2 (%)		97.4 (3.3)	75, 100	98 (95.0, 99.8)		96.8 (3.7)	82, 100	98 (94.8, 99.0)		97.8 (3.1)	75, 100	98 (95.6, 100.0)
Missing of SpO_2	83 (35.0%)				34 (39.1%)				49 (32.7%)			
**Chief complaint at ED**												
Disturbance of consciousness	98 (41.4%)				28 (32.2%)				70 (46.7%)			
Seizure	11 (4.6%)				3 (3.4%)				8 (5.3%)			
Others	53 (22.4%)				15 (17.2%)				38 (25.3%)			
Missing	75 (31.6%)				41 (47.1%)				34 (22.7%)			
**Initial blood sugar level at the ED (mg/dl)**		71.2 (62.7)	9, 353	44 (22.0, 100.0)		37.4 (29.8)	9, 186	32 (17.8, 44.0)		83.3 (66.8)	11, 353	56.5 (26.4, 122.5)
≥ 70	45 (19%)				3 (3.4%)				42 (28.0%)			
50-69	16 (6.8%)				0 (0.0%)				0 (0.0%)			
< 50	80 (33.8%)				34 (39.1%)				62 (41.3%)			
Missing	96 (40.5%)				50 (57.5%)				46 (30.7%)			
**Intervention for hypoglycemia in the ED**	177 (74.7%)				68 (78.2%)				109 (72.7%)			

Glucose administration	177 (100.0%)	68 (100.0%)	109 (100.0%)
**Primary diagnosis at the ED**			
Hypoglycemia	150 (63.3%)	60 (69.0%)	90 (60.0%)
Hypoglycemic attack	56 (23.6%)	25 (28.7%)	31 (20.7%)
Hypoglycemic encephalopathy	2 (0.8%)	1 (1.1%)	1 (0.7%)
**Concurrent conditions**			
Hypothermia	13 (5.5%)	3 (3.4%)	10 (6.7%)
Urinary tract infection	6 (2.5%)	1 (1.1%)	5 (3.3%)
Disturbance of consciousness	5 (2.1%)	0 (0.0%)	5 (3.3%)
Heart failure	4 (1.7%)	0 (0.0%)	4 (2.7%)
Hypokalemia	4 (1.7%)	2 (2.3%)	2 (1.3%)
**Prognosis**			
Discharged home	123 (51.9%)	60 (69.0%)	63 (42%)
Hospitalization	105 (44.3%)	23 (26.4%)	82 (54.7%)
Death in the ED	1 (0.4%)	0 (0.0%)	1 (0.7%)
Missing	8 (3.4%)	4 (4.6%)	4 (2.7%)
**Admission ward**			
General ward	105 (44.3%)	23 (100.0%)	82 (54.7%)

Categorical variables: n (%). Continuous variables: mean, standard deviation (SD) ,min, max, median, Q1 and Q3.

ED: Emergency Department, GCS: Glasgow Coma Scale, EMS: Emergency Medical Services.

Table S4. Missing data-specific characteristics and circumstances of patients with severe hypoglycemia in the prehospital setting

Variables	Missing data of initial BS N= (130)				Missing data of BS at ED N= (96)				Missing data of JCS at ED N= (113)			
	n (%)	Mean (SD)	min, max	Median (Q1, Q3)	n (%)	Mean (SD)	min, max	Median (Q1, Q3)	n (%)	Mean (SD)	min, max	Median (Q1, Q3)
**Age (year)**		69.8 (18.6)	1, 104	74 (61.3, 83.0)		67.5 (20.6)	1.0, 100.0	75.0 (61.8, 83.0)		70.3 (17.8)	8.0, 93.0	75.0 (60.0, 83.0)
**Male**	86 (66.2%)				62 (64.6%)				66 (58.4%)			
**BMI (kg/m^2)**		20.8 (5.0)	10.3, 46.3	20.2 (17.8, 23.1)		21.3 (3.9)	12.7, 27.7	20.5 (18.3, 24.7)		21.5 (4.7)	14.3, 46.3	20.8 (18.4, 23.6)
Missing	29 (22.3%)				20 (20.8%)				22 (19.5%)			
**Past medical history**												
Type 1 diabetes	15 (11.5%)				7 (7.3%)				1 (0.9%)			
Type 2 diabetes	45 (34.6%)				44 (45.8%)				29 (25.7%)			
Diabetes other	5 (3.8%)				5 (5.2%)				4 (3.5%)			
Diabetes associated with pancreatic cancer	3 (2.3%)				4 (4.2%)				3 (2.3%)			
Diabetes secondary to chronic pancreatitis	1 (0.8%)				1 (1.0%)				1 (0.9%)			
Mitochondrial disorder	1 (0.8%)				0 (0.0%)				0 (0.0%)			
Unspecified (e.g., only “diabetes”)	65 (50.0%)				40 (41.7%)				79 (69.9%)			
**Regular use of diabetes medication**	48 (36.9%)				34 (35.4%)				43 (38.1%)			
**Details of medication**	(n=48)				(n=34)				(n=43)			
Insulin	22 (45.8%)				24 (70.6%)				27 (62.8%)			
Biguanide class	8 (16.7%)				7 (20.6%)				7 (16.3%)			
Thiazolidinedione class	3 (6.3%)				2 (5.9%)				4 (9.3%)			

DPP-4 inhibitors	22 (45.8%)	10 (29.4%)	18 (41.9%)
Sulfonylurea class	13 (27.1%)	4 (11.8%)	6 (14.0%)
Glinide class	4 (8.3%)	0 (0.0%)	2 (4.7%)
Alpha-glucosidase inhibitors	12 (25.0%)	8 (23.5%)	9 (20.9%)
SGLT2 inhibitors	9 (18.8%)	11 (32.4%)	16 (37.2%)
GLP-1 receptor agonist	4 (8.3%)	0 (0.0%)	2 (4.7%)
GIP/GLP-1 receptor agonist	0 (0.0%)	0 (0.0%)	0 (0.0%)
**Location**			
Home	102 (78.5%)	76 (79.2%)	91 (80.5%)
Public place	13 (10.0%)	11 (11.5%)	11 (9.7%)
Public road/street/park	7 (5.4%)	6 (6.3%)	4 (3.5%)
Short-term care facility	0 (0.0%)	0 (0.0%)	0 (0.0%)
Lodging facility	0 (0.0%)	0 (0.0%)	1 (0.9%)
Gaming/entertainment venue	0 (0.0%)	0 (0.0%)	0 (0.0%)
Farm/agricultural facility	1 (0.8%)	0 (0.0%)	1 (0.9%)
Retail store	0 (0.0%)	0 (0.0%)	0 (0.0%)
Supermarket	1 (0.8%)	1 (1.0%)	1 (0.9%)
Unknown/unspecified	4 (3.1%)	4 (4.2%)	4 (3.5%)
Residential facility	10 (7.7%)	6 (6.3%)	9 (8.0%)
Workplace	4 (3.1%)	3 (3.1%)	2 (1.8%)
Others/unspecified	1 (0.8%)	0 (0.0%)	0 (0.0%)
**Estimated onset time**			
0:00-5:59	9 (6.9%)	10 (10.4%)	9 (8.0%)
6:00-11:59	27 (20.8%)	17 (17.7%)	18 (15.9%)
12:00-17:59	27 (20.8%)	15 (15.6%)	15 (13.3%)
18:00-23:59	67 (51.5%)	54 (56.2%)	71 (62.8%)

**Dispatch complaint**			
Hypoglycemia	4 (3.1%)	12 (12.5%)	15 (13.3%)
Consciousness disorder	54 (41.5%)	27 (28.1%)	46 (40.7%)
Others	35 (26.9%)	13 (13.5%)	23 (20.4%)
Missing	37 (28.5%)	44 (45.8%)	29 (25.7%)
**Person who discovered the patient or was involved in contacting EMS**			
Self	17 (13.1%)	5 (5.2%)	6 (5.3%)
Family	81 (62.3%)	69 (71.9%)	79 (69.9%)
Spouse	37 (45.7%)	26 (27.1%)	33 (29.2%)
Parent	5 (6.2%)	6 (6.2%)	3 (2.7%)
Sibling	5 (6.2%)	6 (6.2%)	6 (5.3%)
Child	18 (22.2%)	16 (16.7%)	23 (20.4%)
Unknown	16 (19.8%)	15 (15.6%)	14 (12.4%)
Non-family Acquaintance (e.g., relative、acquaintance)	3 (2.3%)	3 (3.1%)	6 (5.3%)
Others （e.g., coworker, care facility staff, daycare service, taxi driver）	28 (21.5%)	19 (19.8%)	22 (19.5%)
Missing	1 (0.8%)	0 (0.0%)	0 (0.0%)
**Prehospital Intervention for hypoglycemia**	30 (23.1%)	53 (55.2%)	60 (53.1%)
**Who**			
Self	6 (20.0%)	1 (1.9%)	4 (6.7%)
Family	5 (16.7%)	5 (9.4%)	4 (6.7%)
Spouse	3 (10.0%)	3 (5.7%)	3 (5.0%)
Child	0 (0.0%)	2 (3.8%)	0 (0.0%)
Unknown	2 (6.7%)	0 (0.0%)	1 (1.7%)
Non-family Acquaintance	0 (0.0%)	0 (0.0%)	0 (0.0%)

EMS team	14 (46.7%)	42 (79.1%)	41 (68.3%)
Others	4 (13.3%)	4 (7.5%)	7 (11.7%)
Missing/unspecified	1 (3.3%)	1 (1.9%)	0 (0.0%)
**When**			
Before EMS arrival	14 (46.7%)	14 (26.4%)	17 (28.3%)
During transport	16 (53.3%)	39 (73.6%)	43 (71.7%)
Missing	0 (0.0%)	0 (0.0%)	0 (0.0%)
**What**			
Oral glucose intake	9 (30.0%)	8 (15.1%)	14 (23.3%)
Intravenous glucose administration	13 (43.3%)	42 (79.1%)	41 (68.3%)
Candy	6 (20.0%)	1 (1.9%)	3 (5.0%)
Juice	1 (3.3%)	1 (1.9%)	1 (1.7%)
Other meals	1 (3.3%)	0 (0.0%)	1 (1.7%)
Missing	0 (0.0%)	1 (1.9%)	0 (0.0%)

Categorical variables: n (%). Continuous variables: mean, standard deviation (SD) ,min, max, median, Q1 and Q3.

BMI: Body Mass Index,

EMS: Emergency Medical Services.

Table S5. Missing data-specific prehospital assessment, intervention, and timeline

Variables	Missing data of initial BS N= (130)				Missing data of BS at ED N= (96)				Missing data of JCS at ED N= (113)			
	n (%)	Mean (SD)	min, max	Median (Q1, Q3)	n (%)	Mean (SD)	min, max	Median (Q1, Q3)	n (%)	Mean (SD)	min, max	Median (Q1, Q3)
**Vital signs on EMS arrival**												
**Japan Coma Scale (JCS)**												

Alert	31 (23.8%)				16 (16.7%)				19 (16.8%)			
Ⅰ-1	16 (12.3%)				4 (4.2%)				12 (10.6%)			
Ⅰ-2	7 (5.4%)				3 (3.1%)				5 (4.4%)			
Ⅰ-3	28 (21.5%)				15 (15.6%)				13 (11.5%)			
Ⅱ-10	13 (10%)				16 (16.7%)				12 (10.6%)			
Ⅱ-20	4 (3.1%)				3 (3.1%)				5 (4.4%)			
Ⅱ-30	3 (2.3%)				5 (5.2%)				9 (8.0%)			
Ⅲ-100	6 (4.6%)				4 (4.2%)				7 (6.2%)			
Ⅲ-200	3 (2.3%)				5 (5.2%)				6 (5.3%)			
Ⅲ-300	13 (10%)				10 (10.4%)				8 (7.1%)			
Missing of JCS	6 (4.6%)				15 (15.6%)				17 (15.0%)			
**Body temperature (BT) (℃)**		35.9 (1.5)	31.2, 40.1	36.2 (35.4, 36.8)		36.1 (1.4)	31.6, 40.1	36.2 (35.9, 36.6)		36.1 (1.3)	31.3, 39.3	36.2 (35.7, 36.6)
Missing of BT	9 (6.9%)				20 (20.8%)				20 (17.7%)			
**Heart rate (HR) (/min)**		87.9 (21.9)	20.0, 180.0	86.0 (75.0, 98.5)		88.4 (23.4)	39.0, 180.0	81.0 (74.5, 100.0)		89.5 (21.7)	39.0, 158.0	86.0 (75.0, 105.0)
Missing of HR	7 (5.4%)				17 (17.7%)				20 (17.7%)			
**Systolic blood pressure (SBP) (mmHg)**		146.1 (36.2)	55.0, 219.0	148.0 (118.0, 174.5)		151.4 (35.7)	75.0, 268.0	150.0 (128.0, 170.0)		151.5 (33.5)	75.0, 235.0	150.0 (126.8, 171.8)
Missing of SBP	19 (14.6%)				26 (27.1%)				29 (25.7%)			
**Diastolic blood pressure (DBP) (mmHg)**		84.9 (25.6)	40.0, 190.0	82.0 (68.0, 98.0)		83.7 (22.0)	42.0, 163.0	83.0 (70.0, 95.2)		87.0 (27.3)	40.0, 190.0	81.0 (69.0, 98.0)
Missing of DBP	21 (16.2%)				28 (29.2%)				30 (26.5%)			
**Respiratory rate (RR) (/min)**		21.2 (5.1)	10.0, 45.0	20.0 (18.0, 24.0)		20.8 (4.7)	10.0, 36.0	20.0 (18.0, 24.0)		21.3 (3.9)	17.0, 36.0	20.0 (18.0, 24.0)
Missing of RR	9 (6.9%)				21 (21.9%)				22 (19.5%)			
**SpO_2 (%)**		94.9 (6.6)	66.0, 100.0	97.0 (95.0, 99.0)		96.2 (5.1)	66.0, 100.0	98.0 (96.0, 99.0)		95.4 (5.9)	66.0, 100.0	98.0 (95.0, 99.0)
Missing of SpO_2	14 (10.8%)				20 (20.8%)				21 (18.6%)			

**Initial blood sugar level on EMS arrival (mg/dl)**		-	-	-		35.5 (36.2)	10.0, 285.0	27.0 (20.0, 37.0)		37.3 (38.3)	14.0, 285.0	29.0 (20.0, 40.0)
≥ 70	0 (0.0%)				2 (2.1%)				3 (2.7%)			
50-69	0 (0.0%)				5 (5.2%)				3 (2.7%)			
< 50	0 (0.0%)				53 (55.2%)				47 (41.6%)			
Missing	130 (100%)				36 (37.5%)				60 (53.1%)			
**Activation of glucose administration protocol by emergency team**	109 (83.8%)				67 (69.8%)				81 (71.7%)			
**When**												
From EMS arrival to departure	0 (0.0%)				5 (7.5%)				2 (2.5%)			
During transport	14 (12.8%)				37 (55.2%)				39 (48.1%)			
Missing	95 (87.2%)				25 (37.3%)				40 (49.4%)			
**What**												
Glucose administration	14 (12.8%)				41 (61.2%)				40 (49.4%)			
Missing	95 (87.2%)				26 (38.8%)				41 (50.6%)			
**Timeline regarding hypoglycemia (min)**												
From EMS call to EMS arrival		7.6 (3.2)	1, 16	7 (5.0, 9.0)		9.0 (3.5)	3.0, 19.0	8.0 (7.0, 11.0)		7.4 (3.5)	1.0, 19.0	6.0 (5.0, 9.0)
From EMS call to hospital arrival		37.7 (9.2)	18, 67	38 (32.0, 43.8)		43.7 (12.5)	19.0, 74.0	41.5 (35.8, 51.2)		39.2 (10.8)	18.0, 74.0	39.0 (32.0, 46.0)
**Time from emergency call to arrival on scene, min**												
On-site duration (EMS arrival – EMS departure)		17.4 (6.8)	0, 43	16.5 (12.0, 22.0)		20.4 (8.8)	6.0, 57.0	19.0 (15.0, 24.2)		19.1 (8.2)	6.0, 57.0	18.0 (13.0, 24.0)
Total consultation time		21.2 (56.9)	0, 355	4 (3.0, 13.0)		7.5 (10.9)	0.0, 50.0	4.0 (2.0, 7.0)		8.7 (12.0)	0.0, 57.0	5.0 (3.2, 8.0)
Total transport time (EMS call to hospital arrival)		36.3 (9.3)	17, 67	36 (30.0, 42.0)		42.7 (12.8)	18.0, 74.0	40.0 (34.0, 50.2)		37.7 (11.0)	17.0, 74.0	37.0 (30.0, 44.0)

Categorical variables: n (%). Continuous variables: mean, standard deviation (SD) ,min, max, median, Q1 and Q3.

GCS: Glasgow Coma Scale, EMS: Emergency Medical Services.

Table S6. Missing data-specific details of management of severe hypoglycemia in the emergency department

Variables	Missing data of initial BS N= (130)				Missing data of BS at ED N= (96)				Missing data of JCS at ED N= (113)			
	n (%)	Mean (SD)	min, max	Median (Q1, Q3)	n (%)	Mean (SD)	min, max	Median (Q1, Q3)	n (%)	Mean (SD)	min, max	Median (Q1, Q3)
**Vital signs at the ED**												
Japan Coma Scale (JCS)												
Alert	21 (16.2%)				12 (12.5%)				0 (0.0%)			
Ⅰ-1	11 (8.5%)				9 (9.4%)				0 (0.0%)			
Ⅰ-2	5 (3.8%)				4 (4.2%)				0 (0.0%)			
Ⅰ-3	10 (7.7%)				6 (6.2%)				0 (0.0%)			
Ⅱ-10	6 (4.6%)				2 (2.1%)				0 (0.0%)			
Ⅱ-20	5 (3.8%)				0 (0.0%)				0 (0.0%)			
Ⅱ-30	3 (2.3%)				1 (1.0%)				0 (0.0%)			
Ⅲ-100	2 (1.5%)				1 (1.0%)				0 (0.0%)			
Ⅲ-200	2 (1.5%)				4 (4.2%)				0 (0.0%)			
Ⅲ-300	5 (3.8%)				2 (2.1%)				0 (0.0%)			
Missing of JCS	60 (46.2%)				55 (57.3%)				113 (100.0%)			
Body temperature (BT) (℃)		35.4 (1.6)	31.1, 38.9	35.8 (34.5, 36.5)		35.4 (1.9)	28.0, 38.9	35.8 (34.8, 36.5)		35.5 (1.4)	31.1, 38.3	35.8 (34.6, 36.4)
Missing of BT	35 (26.9%)				41 (42.7%)				56 (49.6%)			
Heart rate (HR) (/min)		86.9 (21.1)	20.0, 166.0	85.0 (74.0, 97.0)		81.0 (19.1)	36.0, 144.0	79.0 (70.0, 91.2)		85.2 (20.9)	36.0, 166.0	82.0 (73.0, 96.0)
Missing of HR	33 (25.4%)				40 (41.7%)				55 (48.7%)			

Systolic blood pressure (SBP) (mmHg)		138.2 (39.7)	54.0, 222.0	137.0 (105.0, 170.0)		151.8 (36.3)	85.0, 250.0	146.0 (127.2, 175.8)		143.6 (34.8)	65.0, 229.0	142.0 (118.0, 170.0)
Missing of SBP	33 (25.4%)				42 (43.8%)				54 (47.8%)			
Diastolic blood pressure (DBP) (mmHg)		79.6 (22.1)	41.0, 135.0	80.5 (61.0, 93.2)		84.4 (19.1)	50.0, 166.0	84.5 (71.5, 93.8)		82.9 (21.8)	48.0, 135.0	80.5 (68.0, 98.0)
Missing of DBP	34 (26.2%)				42 (43.8%)				54 (47.8%)			
Respiratory rate (RR) (/min)		20.4 (5.7)	11.0, 40.0	20.0 (16.8, 24.0)		19.5 (5.0)	12.0, 40.0	18.0 (15.8, 23.2)		20.6 (5.4)	12.0, 40.0	20.0 (17.0, 24.0)
Missing of RR	38 (29.2%)				44 (45.8%)				55 (48.7%)			
SpO_2 (%)		97.2 (3.5)	75.0, 100.0	98.0 (97.0, 99.0)		97.2 (3.5)	82.0, 100.0	98.0 (96.0, 99.0)		98.0 (2.2)	91.0, 100.0	99.0 (97.0, 100.0)
Missing of SpO_2	45 (34.6%)				47 (49.0%)				57 (50.4%)			
**Chief complaint at ED**												
Disturbance of consciousness	54 (41.5%)				29 (30.2%)				50 (44.2%)			
Seizure	8 (6.2%)				3 (3.1%)				5 (4.4%)			
Others	31 (23.8%)				20 (20.8%)				29 (25.7%)			
Missing	37 (28.5%)				44 (45.8%)				29 (25.7%)			
**Initial blood sugar level at the ED (mg/dl)**		61.8 (60.7)	9.0, 353.0	40.0 (26.2, 68.0)		-	-	-		86.9 (70.7)	12.0, 243.0	51.0 (34.0, 155.5)
≥ 70	23 (17.7%)				0 (0.0%)				22 (19.5%)			
50-69	10 (7.7%)				0 (0.0%)				7 (6.2%)			
< 50	61 (46.9%)				0 (0.0%)				29 (25.7%)			
Missing	36 (27.7%)				96 (100.0%)				55 (48.7%)			
**Intervention for hypoglycemia in the ED**	109 (83.8%)				67 (69.8%)				81 (71.7%)			
Glucose administration	109 (100.0%)				67 (100.0%)				81 (100.0%)			

Others	0 (0.0%)	0 (0.0%)	0 (0.0%)
**Primary diagnosis at the ED**			
Hypoglycemia	83 (63.8%)	42 (43.8%)	43 (38.1%)
Hypoglycemic attack	21 (16.2%)	28 (29.2%)	26 (23.0%)
Hypoglycemic encephalopathy	2 (1.5%)	1 (1.0%)	1 (0.9%)
**Concurrent conditions**			
Hypothermia	12 (9.2%)	2 (2.1%)	9 (8.0%)
Urinary tract infection	4 (3.1%)	0 (0.0%)	2 (1.8%)
Disturbance of consciousness	4 (3.1%)	1 (1.0%)	1 (0.9%)
Heart failure	3 (2.3%)	2 (2.1%)	2 (1.8%)
Hypokalemia	3 (2.3%)	1 (1.0%)	2 (1.8%)
**Prognosis**			
Discharged home	55 (42.3%)	60 (62.5%)	57 (50.4%)
Hospitalization	70 (53.8%)	34 (35.4%)	51 (45.1%)
Death in the ED	1 (0.8%)	0 (0.0%)	0 (0.0%)
Missing	4 (3.1%)	2 (2.1%)	5 (4.4%)
**Admission ward**			
ICU	0 (0.0%)	0 (0.0%)	0 (0.0%)
General ward	70 (53.8%)	34 (35.4%)	51 (45.1%)

Categorical variables: n (%). Continuous variables: mean, standard deviation (SD) ,min, max, median, Q1 and Q3.

ED: Emergency Department, GCS: Glasgow Coma Scale, EMS: Emergency Medical Services.
