# Supplementary figures and images for: Hypoglycemic Events Focusing on Situational Factors, Bystander Identification, and Prehospital Management
Source: J Clin Med. 2026 Apr 5;15(7):2746. doi: 10.3390/jcm15072746 (PMC13073431; doi:10.3390/jcm15072746)

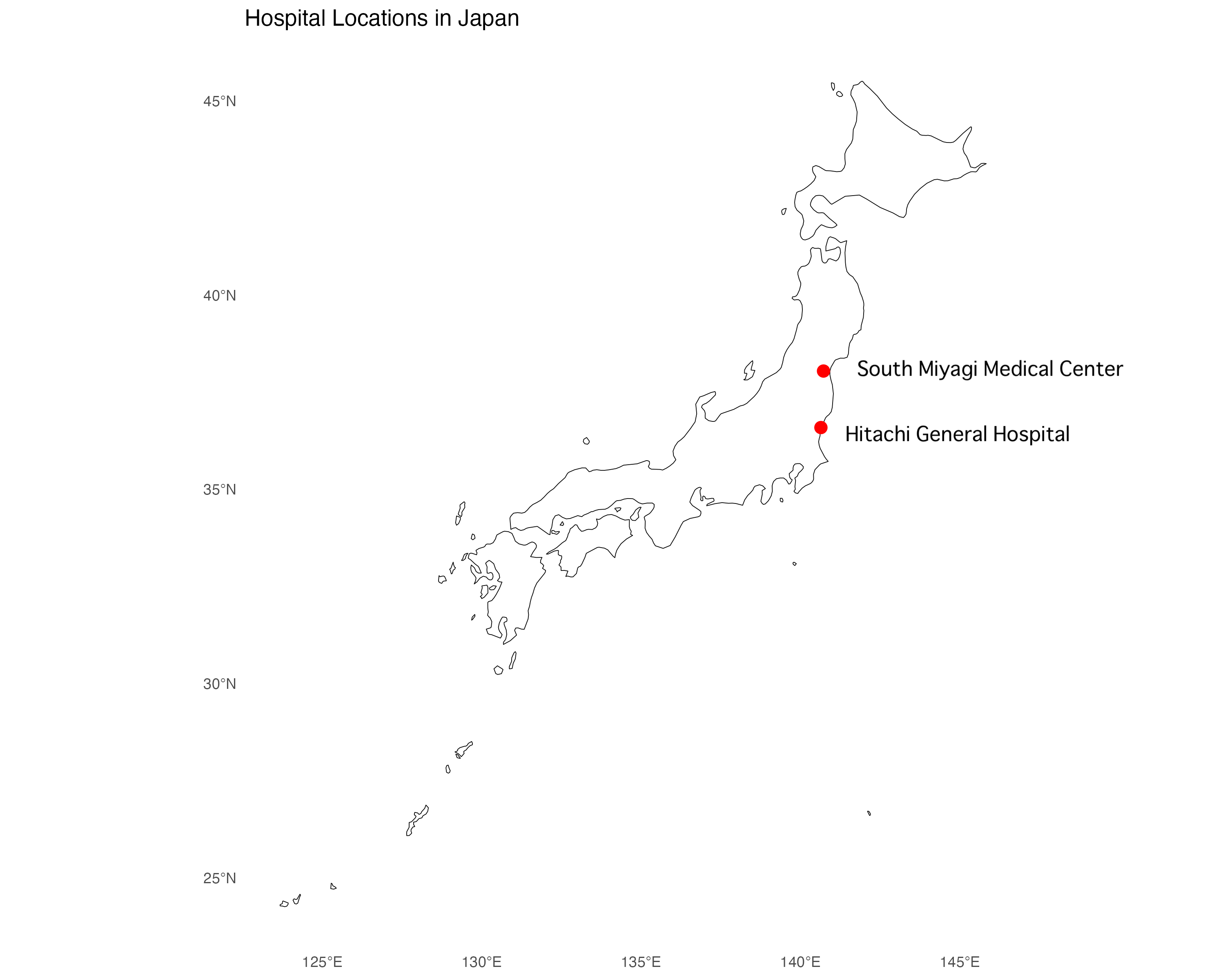

Supplement: Supplementary file 1 [file jcm-15-02746-s001.zip › Supplementary_Figure.tiff]
